# Supplementary figures and images for: Morphological and Molecular Characterization of Lema bilineata (Germar), a New Alien Invasive Leaf Beetle for Europe, with Notes on the Related Species Lema daturaphila Kogan & Goeden
Source: Insects. 2020 May 11;11(5):295. doi: 10.3390/insects11050295 (PMC7290676; doi:10.3390/insects11050295)

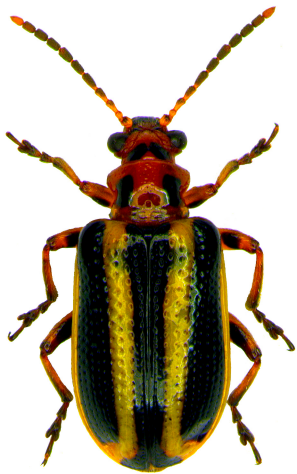

a

2 mm

---

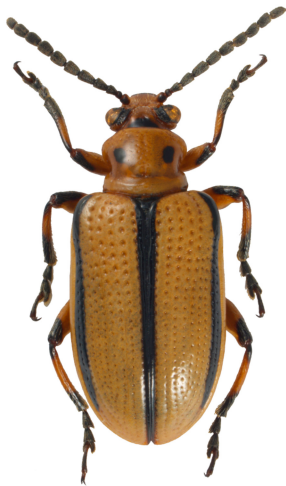

b

Supplement: Supplementary file 1 [file insects-11-00295-s001.pdf]
